# Muscular and cardiorespiratory fitness are associated with health-related quality of life among young adult men

**DOI:** 10.1186/s12889-020-08969-y

**Published:** 2020-06-03

**Authors:** Kaija Appelqvist-Schmidlechner, Jani P. Vaara, Tommi Vasankari, Arja Häkkinen, Matti Mäntysaari, Heikki Kyröläinen

**Affiliations:** 1 Finnish Institute for Health and Welfare / Centre for Military Medicine, Helsinki, Finland; 2 The Department of Leadership and Military Pedagogy, National Defence University, Helsinki, Finland; 3 UKK institute for Health Promotion, Tampere, Finland; 4 Department of Physical Medicine and Rehabilitation, Health Sciences / Central Hospital of Central Finland, University of Jyväskylä, Jyväskylä, Finland; 5 Centre for Military Medicine, Helsinki, Finland; 6 Department of biology of Physical Activity, University of Jyväskylä, Jyväskylä, Finland

**Keywords:** Health-related quality of life, Physical fitness, Mental health, Physical activity, Young men, Cardiorespiratory fitness, Muscular fitness

## Abstract

**Background:**

Despite numerous studies providing evidence for positive effects of physical activity and physical fitness, evidence for association between physical fitness and health-related quality of life (HRQoL) in young adults is limited. The aim of the present cross-sectional study was to investigate the association of cardiorespiratory and muscular fitness with HRQoL from the perspective of its physical and mental components among young adult Finnish males.

**Methods:**

The sample consisted of 754 men, with the mean age of 26 years (SD 6.7 years), who participated in the military refresher training. HRQoL was measured using the Finnish RAND 36-item health survey. Cardiorespiratory fitness was determined by a bicycle ergometer test, and muscular fitness by various tests measuring maximal strength and muscular endurance. Logistic regression modelling was used to compare low, moderate and high physical and mental component of HRQoL scores to the respective levels of muscular and cardiorespiratory fitness.

**Results:**

The findings of the adjusted (age, educational level, marital status, employment status, smoking, use of alcohol and BMI) analysis showed that cardiorespiratory and muscular fitness are positively associated with both physical and mental components of HRQoL. In terms of the physical component of HRQoL, even a moderate fitness level was positively associated with better HRQoL. In terms of the mental component of HRQoL, the impact was seen only in the group with the highest fitness level.

**Conclusions:**

The findings suggest a positive contribution of physical fitness to mental health and highlight the importance of both muscular and cardiorespiratory fitness in the promotion of HRQoL. Even lighter forms of physical activity that result in moderate physical fitness could contribute to the physical component of HRQoL. In terms of the mental component of HRQoL, higher levels of physical fitness may be needed to gain higher levels of HRQoL among young males.

## Background

Physical activity is known to have a positive association with health-related quality of life (HRQoL [1, 2];). Evidence regarding the relationship between the level of physical fitness and HRQoL, however, is limited. To date, studies related to this topic have most commonly focused on specific target groups with specific health concerns [3–6] or conditions [7, 8] finding a positive relationship between physical fitness and HRQoL. Only few studies have explored this association in the general adult population [9–11], but not separately for muscular and cardiorespiratory fitness.

Health-related quality of life is a multidimensional concept that encompasses physical, mental, emotional and social functioning [12]. It relates to a person’s self-perceived health in terms of well-being and functionality in different areas of life, such as physical well-being and functioning, emotional well-being, self-esteem, social functioning and family relations [13]. The concept has gained much attention in the past few decades, as it has been found to be a stronger predictor of mortality and morbidity than many other objective measures of health [14, 15]. HRQoL has been shown to be associated with various socioeconomic factors and variables related to health behaviour, such as BMI, use of alcohol and smoking [16].

Physical fitness is widely recognized as a powerful marker of health-related outcomes and as an important determinant of current and future health status [17]. The health-related components of physical fitness are commonly classified as cardiorespiratory fitness, muscular strength and endurance, body composition and flexibility, balance, agility, reaction time and power [18]. In previous studies, physical fitness was typically explored from the perspective of cardiorespiratory fitness [10, 11, 19, 20], muscular fitness [21, 22] or a combination of them [9, 11, 23] and was determined by several field tests. In young adulthood, low muscular strength has been found to be an emerging risk factor for major causes of death, such as suicide and cardiovascular diseases [21] and to be associated with higher levels of stress [24], lower level of mental well-being [22, 24] and increased health-risk behaviour [22]. Similarly, reduced cardiorespiratory fitness has been found to associate with higher levels of depressive symptoms [25, 26], stress [24] and stress-related exhaustion [27] as well as lower levels of mental well-being [24]. In terms of HRQoL, a positive association with physical fitness has been found in the samples of middle aged [11] and young men [9, 10] from the perspective of cardiorespiratory and muscular fitness.

Despite numerous studies providing evidence for various positive effects of physical activity and physical fitness, evidence for association between physical fitness and HRQoL in the general population is limited. Due to the lack of evidence, the role of physical fitness in contributing to a better HRQoL remains unclear. Very little is also known about how different components of fitness contribute to HRQoL in young men, who can be seen as a hard-to-reach group in health surveys. Knowledge on this topic is relevant, for example, for the development of sport-based interventions that promote both physical and mental health as well as quality of life in young adult men.

The aim of the present cross-sectional study was to investigate the association of objectively measured cardiorespiratory and muscular fitness with HRQoL among young adult Finnish men. The association was explored from the perspective of physical and mental components of HRQoL. It was expected that better cardiorespiratory and muscular fitness are both associated with better HRQoL.

## Methods

The study is part of the Finnish Reservist 2015 study which aimed to investigate the functional capacity and health of Finnish reservists. The participants were young adult men who were called up to the military refresher training organized by the Finnish Defence Forces. In Finland, the Defence Forces are based on a universal male conscription. According to the law of national defence, conscription time starts at the age of 18, and compulsory military service must be performed by the age of 30 years. Each year, 70–75% of all young Finnish men perform their military service (~ 20,000 men). After the military service, they continue with their normal civilian lives, but as reservists, they can be called up to a military refresher training lasting 4–10 days.

The participants were informed about the study in the military refresher training call-up letter. The data for the present study were gathered at the beginning of seven military refresher training courses which were carried out in 2015 in different counties around Finland. The data were collected with the help of several fitness tests and a self-administered questionnaire. Participation in the study was voluntary and, of 823 course participants, 784 participated in the study. Altogether, 754 men with the mean age of 26 years (SD 6.7 years) participated in the fitness tests and comprised the sample of this study. All participants signed a written consent form. The study was approved by the ethical committees of the Central Finland Health Care District, and the Headquarters of the Finnish Defence Forces (AM5527).

### Measurements

*Health-related quality of life* (HRQoL) was measured using the Finnish RAND 36-item health survey [28]. It contains the following eight dimensions: physical functioning, physical role functioning, emotional role functioning, vitality, mental health, social role functioning, bodily pain and general health perceptions. These eight dimensions can be aggregated into two summary scores: the physical (including physical functioning, physical role functioning, bodily pain and general health) and mental (including emotional role functioning, vitality mental health and social role functioning) component summary scores. The responses are given in a six-point scale. The calculation of the scores was made in a two-step process. First, numeric values were recoded per the scoring key and transformed into a 0 to 100 scale higher scores indicating higher HRQoL. Then, items in the same dimensions were averaged together to create the scores for the eight dimensions.

*Physical fitness was* measured by six consecutive tests in the following order: maximal standing long jump, isometric maximal force of lower (leg press) and upper extremities (bench press), cardiorespiratory fitness (maximal oxygen uptake), push-ups and sit-ups in 1 min. A recovery period lasting at least 30 min after cardiorespiratory fitness tests was allowed prior to the beginning of muscular endurance tests.

*Standing broad jump test* was used to assess explosive force production of lower extremities [29]. Prior to testing, which was performed on a specifically designed gym mat, the participants were instructed of the correct technique, and they performed a warm-up and several practice jumps. The 10-min warm-up included of calisthenics exercises (x-jumps, push-ups, sit-ups, squats, planks and countermovement jumps). The participants were instructed to jump (horizontally) forward as far as possible from a standing position without falling backward upon bilateral landing. Three trials were completed with each interspersed by a 1-min rest period. The distance was measured with 1 cm precision.

*Maximal isometric force* was measured with horizontal bench press and leg press using dynamometers. Knee angle was set to 107° in leg press and hands were placed on a handle grip. In the maximal bench press test, the participants were in supine position with their backs flat on a bench and feet flat on the floor. Elbow and shoulder were positioned at 90°. A warm-up consisted of at least 2 submaximal sets. Three trials were performed when assessing maximal performance using a 30-s recovery period. The best performance was included in the analysis. The participants were advised to produce maximal force as fast as possible for 3 sec. The participants were verbally encouraged during the maximal efforts. The repeatability of this test has been reported to be high [30].

*Muscular endurance* tests consisted of push-ups and sit-ups (repetitions/minute). The push-up test measures arm and shoulder extensor muscle performance. At the start, the participants laid face down on the floor, feet parallel at pelvis to shoulder width and hands positioned so that thumbs could reach the shoulders while other fingers were pointing forward. Before the start of the test, the participants were instructed to extend arms to the starting position and keep the feet, trunk, and the shoulders in the same line during the test performance. A successful repetition was counted when the participant lowered his torso with flexing arms to an elbow angle of 90° and returned to the starting position by extending his arms. Sit-up test measured performance of abdominal and hip flexor muscles. At the start, the participant laid on a back while legs were supported from the ankles by an assistant. The knees were flexed at the angle of 90°, elbows pointing upward while fingers crossed behind the back of the head. A successful repetition was counted when the participant lifted his upper body from the starting position and brought elbows to the knee-level. The result was expressed as a number of consecutive successful repetitions during 1 min. A recovery period lasting 5-min was allowed between the tests. Correct technique was demonstrated to participants before each test and only the trials with adequate technique were accepted. The test-retest reliability of push-up and sit-up tests has been reported to be high among young adults and middle-aged adults [31, 32].

*Cardiorespiratory fitness* (VO_2_max) was assessed using an indirect graded cycle ergometer test (Ergoline 800S, Ergoselect 100 K, Ergoselect 200 K, Bitz, Germany) until exhaustion. A progressive protocol started initially with a 50 W power output and was increased 25 W every 2 min until exhaustion. Heart rate (HR) was recorded throughout the test (Polar Vantage NV or S610, S710, or S810, Kempele, Finland). Predicted VO_2_max (FitWare, Mikkeli, Finland), based on maximal power produced in the end of the test, was determined according to the following equation: VO_2_max (ml·kg^− 1^·min^− 1^) = 12.35 × Pmax/kg + 3.5, where the Pmax is the highest work rate (maximal power) achieved during the test as watts, and body mass as kilograms). The intra class correlation has been reported to be high with this method [33].

Results from each muscular fitness test were divided into three categories with the help of z-scores and tertiles indicating low, moderate and high level of muscular fitness in each test. Sum score of these 5 tests were calculated using z-scores. Finally, the sum score was divided into tertiles indicating low, moderate and high level of muscular fitness. Cardiorespiratory fitness and scores of HRQoL were similarly divided into three tertiles (low, moderate, high level).

*Covariates* used in this study were body mass index (underweight/normal/overweight/obesity or severe obesity), smoking (yes/no), use of alcohol (weekly use yes/no), educational level (primary/secondary/higher education), employment status (in employment or education yes/no) and marital status (married or cohabitation yes/no) since they have been shown to be associated with HRQoL [16]. For measuring BMI, height and weight were measured from each study participants. BMI was classified into four categories: underweight < 18,50, normal 18,50-24,99, overweight 25,00–29,99, obesity/severe obesity ≥30. Use of alcohol and cigarettes as well as sociodemographic background including age, educational level, employment and marital status were determined by a questionnaire.

### Statistics

The scores for each of the eight dimensions of RAND 36, and combined physical and mental component scores, were grouped together by low, moderate or high level of muscular and cardiorespiratory fitness, and the mean scores of each fitness level are presented in Table 1. The statistical significance of the physical and mental component score differences between muscular and cardiorespiratory fitness levels was calculated using the Kruskal Wallis -test (Table 1). The association between physical fitness and HRQoL, in terms of physical and mental component scores of RAND 36, was then explored with the help of logistic regression analyses. For each muscular and cardiorespiratory fitness level, odds ratios and 95% confidence intervals (CI) for low compared to moderate or high scores in the physical and mental component of HRQoL were calculated (Table 2.). Unadjusted and fully adjusted models are presented. Age, educational level (primary, secondary, high school), employment status (employment or education/not in employment or education), marital status (married or cohabitation/single), use of alcohol (weekly use yes/no), smoking and BMI (categories 1–4 presented above) were used as covariates in the fully adjusted model. Analyses were performed without imputing missing values. The level of statistical significance was set to *p* < 0.05.

**Table 1:** Mean scores for the eight domains of RAND 36 and the overall physical and mental components by low, moderate and high level in muscular and cardiorespiratory fitness

	n	low	moderate	high	p ^a^
**Muscular fitness**					
Physical component score	698	86.16	89.06	89.98	<.001
Mental component score	697	77.77	81.65	82.75	0.027
Physical functioning	719	95.54	98.10	98.67	
Physical role functioning	703	94.96	97.40	95.50	
Emotional role functioning	705	82.70	90.19	88.98	
Vitality	716	64.16	69.57	70.87	
Mental health	720	74.80	77,92	80.07	
Social role functioning	716	88.92	88.88	91.12	
Bodily pain	719	84.46	84.47	84.72	
General health perceptions	717	69.54	75.57	81.32	
**Cardiorespiratory fitness**					
Physical component score	707	85,32	88,88	90,74	<.001
Mental component score	703	78,27	81,47	82,42	0.156
Physical functioning	728	94,81	98,76	98,89	
Physical role functioning	712	94,14	95,39	98,02	
Emotional role functioning	713	84,72	87,84	89,72	
Vitality	723	64,28	68,68	70,39	
Mental health	728	74,89	78,85	79,14	
Social role functioning	725	88,73	89,93	89,85	
Bodily pain	728	83,28	84,54	85,00	
General health perceptions	726	69,05	76,80	80,53	

^a^ Statistical significance of differences between the fitness categories measured with Kruskal Wallis -test

**Table 2:** Odds ratios (OR) and 95% confidence intervals (CI) separately for low compared to moderate or high scores in the physical and mental component summary of RAND 36 by low, moderate and high levels of muscular and cardiorespiratory fitness

	Low compared to moderate or high HRQoL Physical component				Low compared to moderate or high HRQoL Mental component			
	unadjusted		fully adjusted ^a^		unadjusted		fully adjusted ^a^	
	OR	CI (95%)	OR	CI (95%)	OR	CI (95%)	OR	CI (95%)
Muscular fitness								
low	ref		ref		ref.		ref.	
moderate	0.63	0.43–0.92*	0.66	0.44–0.98*	0.69	0.47–1.01	0.76	0.51–1.15
high	0.44	0.30–0.64***	0.47	0.31–0.72***	0.56	0.38–0.82**	0.61	0.40–0.93*
Cardiorespiratory fitness								
low	ref.		ref.		ref.		ref.	
moderate	0.45	0.31–0.66***	0.41	0.27–0.62***	0.69	0.47–1.01	0.65	0.42–1.00*
high	0.34	0.23–0.50***	0.34	0.21–0.54***	0.56	0.38–0.82**	0.48	0.30–0.76**

^a^Adjusted for age, educational level, marital status, employment status, smoking, use of alcohol and BMI

***p-value <.001, ** *p*-value<.01, *p-value <.05

## Results

The sample consisted of 754 young men with the mean age of 26 years (SD 6.7 years). Most of them (75%) had a secondary level education, 6% primary and 19% higher education. Almost half (45%) of the men participating in the study was married or lived in a partnership. One third (32%) smoked daily and 81% used alcohol weekly. Half of them (51%) had normal weight, 2% were underweight, one third (34%) overweight and 12% obese.

The differences in unadjusted mean scores of HRQoL components between fitness levels were statistically significant for both muscular and cardiorespiratory fitness (Table 1). The descriptive mean values for each dimension of HRQoL in the low, moderate and high fitness categories are presented in Table 1. Individuals with moderate or high muscular fitness levels had better mean scores of HRQoL than individuals with low fitness levels in both physical (*p* < .001) and mental component of HRQoL (*p* = 027). Better cardiorespiratory fitness associated only with the physical component of HRQoL (p < .001), no association was found between cardiorespiratory fitness and mental components of HRQoL.

The logistic regression analysis showed a positive association between physical fitness and both physical and mental components of HRQoL even if adjusted for age, educational level, marital status, employment status, smoking, use of alcohol and BMI (Table 2). In terms of the physical component of HRQoL, even belonging to the group of moderate fitness level in muscular and cardiorespiratory fitness seemed to associate with a higher quality of life. In terms of the mental component of HRQoL, only high fitness levels in muscular and cardiorespiratory fitness seemed to associate with a higher HRQoL by a statistically significant degree.

After further adjustment for cardiorespiratory fitness, the association between muscular fitness and mental health attenuated to non-significant. However, the association between cardiorespiratory fitness and mental health remained statistically significant after further adjustment for muscular fitness in terms of physical (OR 0.41, 95% CI 0.24–0.69) and mental components (OR 0.51, 95% CI 0.30–0.87) of HRQoL.

## Discussion

The present study showed that a higher fitness level - from the perspective of both muscular and cardiorespiratory fitness - is associated with a higher HRQoL in young men. Similar associations were also found in the sample of another similar cross-sectional study by Häkkinen et al. [9], but without examining muscular and cardiorespiratory fitness separately. Previous studies – mainly with incomparable samples - have particularly highlighted the importance of muscular fitness on HRQoL, for example, among women with autoimmune diseases [4] and in early post-menopause [8] as well as among children [34]. Evidence of cardiorespiratory fitness contributing to the physical well-being dimension of HRQoL has been found in adolescents [35], as well as in young [10] and middle-aged men [11].

The results of the adjusted (age, educational level, marital status, employment status, smoking, use of alcohol and BMI) analysis showed that a higher fitness level is associated with both physical and mental components of HRQoL. This finding is consistent with the previous study on young men in the United States navy of Sloan et al. [10], but partly inconsistent with the study on women with chronic conditions by Gavilan-Carrera [4] which suggested that fitness levels can only predict physical components of HRQoL. The present study allows suggestions of a positive association also between mental components of HRQoL and physical fitness. The mental health dimension of RAND 36 is a widely used and recognized instrument for measuring the status of mental health (The Mental Health Inventory, MHI-5, [36]. According to the unadjusted mean scores of this dimension, mental health seemed to be better among men with the highest fitness level compared to the men with the lowest fitness level in terms of both muscular and cardiorespiratory fitness.

As previous research on physical fitness has mainly focused on mental health outcomes rather than quality of life, there is much – partly controversial - evidence for an association between physical fitness and mental health. Namely, numerous studies have shown – in line with the present findings - a positive association between physical fitness and mental health [19, 37–41]. There is evidence of the important role of cardiorespiratory fitness, particularly, in relation to mental health. According to a systematic review and meta-analysis on the relationship between cardiorespiratory fitness and common mental disorders by Kandola et al. [20], low and medium cardiorespiratory fitness was found to be associated with a greater risk of common mental health disorders, showing a dose-dependent relationship with common mental health disorders. The authors suggest that promoting cardiorespiratory fitness could be useful for preventing common mental disorders. Further, there is evidence that individuals with higher levels of cardiorespiratory fitness have reduced odds for generalized anxiety, panic and depressive symptoms [19, 39, 41], have lower depression symptom severity [19, 37] and higher emotional well-being [37], and that cardiorespiratory fitness may protect against the onset of depression [26, 38] which strengthens the theory of cardiovascular contribution to the aetiology of depression. In a Finnish study on young men, Kettunen et al. [24] found that both cardiorespiratory and muscular fitness together with high leisure time physical activity were associated with low stress and high mental resources. Similar results were also found by Bennie et al. [42, 43] in a study suggesting that both aerobic and muscle-strengthening exercise are associated with a lower prevalence of depression in the adult population.

Despite numerous studies showing a positive association between physical fitness and mental health, some have provided contradictory findings on this relationship. The birth cohort study on young adults at the age of 31 by Suija et al. [25] showed that only muscular fitness was negatively associated with depressive symptoms in both sexes. Similar findings were also found in a longitudinal study by Lindegård et al. [27] who found no association between cardiorespiratory fitness level and anxiety, depression or sleep disturbances in women diagnosed with stress-related exhaustion disorders. However, they did find a positive association between the level of cardiorespiratory fitness and reduced symptoms of stress-related exhaustion over time. The best improvements over time were seen in individuals having a medium cardiorespiratory fitness level. In the present study, the findings showed that, in terms of the physical component of HRQoL, even a moderate fitness level was associated with better HRQoL. In terms of the mental component of HRQoL, the impact on HRQoL was only seen in the group of the highest fitness level. Thus, it seems that even moderate physical fitness level, either inherited or improved by physical activity behaviour, could contribute to the physical component of HRQoL. In terms of the mental component of HRQoL, a higher level of physical fitness may be needed. Interestingly, in the present study, after mutual adjustments for fitness variables, cardiorespiratory fitness remained significantly associated with HRQoL while muscular fitness did not. This finding indicates that cardiorespiratory fitness, independent of muscular fitness, may have a stronger association with HRQoL, but not vice versa.

The mechanisms behind the interaction between physical fitness and HRQoL are known to be diverse and complex [44]. Physical fitness is described to “induce positive psychological and physiological benefits, blunt stress reactivity, protect against potentially adverse behavioural and metabolic consequences of stressful events and prevent many chronic diseases” [44 , p.^1^] which likely also increase HRQoL. Due to increased functional capacity as a result of better physical fitness gained through physical activity, the capacity to live a full, creative and productive life will be supported. One of the mechanisms behind the interaction may also be a more positive body image as a result of physical fitness, contributing particularly on the mental component of HRQoL [45]. However, more evidence of the potential underlying mechanisms is needed.

### Strengths and limitations

Despite the cross-sectional nature of the present study, it provides unique knowledge about the association between objectively measured physical fitness and HRQoL in young Finnish men – commonly seen as a hard-to-reach group in health surveys – by exploring muscular and cardiorespiratory fitness separately. One notable strength of the present study is that physical fitness, in terms of maximal aerobic capacity and muscular fitness, was measured objectively using various widely used and recognized tests.

HRQoL was measured with a validated and widely recognized instrument. In the present study, the difference in scores between fitness groups was commonly 3–7 points, in the general health perceptions section even over 11 points. Differences in some sections seemed quite remarkable compared to previous studies [9], but it is difficult to estimate the clinical importance of these differences despite the statistical significance, thus, is the difference in a score worthwhile or important. Researcher have various opinions about the minimally clinically important differences for the RAND-36 scores (typically set in the range of 3 to 5 points), and caution is recommended when interpreting specific range of points of the scores as minimally clinical important. Even smaller differences may be clinically important – especially in intervention studies [46].

The sample can be seen as a geographically representative sample of Finnish young men. However, the sample consisted of healthy young men who were capable of completing their military service and participating in the military refresher course organized for reservists. As young men exempted from the service are known to have more psychosocial problems than men completing their service [47], it can be assumed that men with various mental and psychosocial problems are under-represented in the sample. Further, some of the reservists were unable to participate in the refresher course due to personal, social or health reasons or because they lived abroad. Without background knowledge of these reservists, it is difficult to estimate the effects of this non-participation on the results. However, the sample can be seen as a relatively good representation of healthy Finnish young men. Due to the cross-sectional study design, causality of the findings cannot be established.

## Conclusions

The present study showed that higher levels of both muscular and cardiorespiratory fitness are associated with higher HRQoL, with respect to both physical and mental components of HRQoL. The findings suggest a positive contribution of physical fitness to mental health and highlight the importance of both muscular and cardiorespiratory fitness while promoting HRQoL. In terms of the physical component of HRQoL, even moderate fitness levels were associated with better HRQoL. In terms of the mental component of HRQoL, the impact was seen only in the group of highest fitness levels. Thus, it seems that even lighter forms of physical activity that result in moderate physical fitness could contribute to the physical component of HRQoL. In terms of the mental component of HRQoL, the findings suggest that higher levels of physical fitness may be needed to gain higher levels of HRQoL in young men. Future studies should investigate potential differences between the genders in order to develop and provide gender specific sport-based programmes and interventions for promotion of both physical and mental health.

## Data Availability

The datasets generated and analysed during the current study are not publicly available but can be requested from the corresponding author with permission the Headquarters of the Finnish Defence Forces and University of Jyväskylä on reasonable request.

## Abbreviations

BMI: Body mass index
CI: Confidence interval
HR: Heart rate
HRQoL: Health related quality of life
kg: Kilogram
ml: Millilitre
OR: Odds ratio
p: *P*-value
Pmax: Maximal power
ref.: Reference
SD: Standard deviation
VO_2_max: Maximal oxygen uptake
W: Watt
